# Thyrotoxic Periodic Paralysis as a Presentation of Thyrotoxicosis: A Case Report and review of the literature

**DOI:** 10.31138/mjr.29.1.46

**Published:** 2018-03-19

**Authors:** Ali Abdul-Rahman Younis

**Affiliations:** Rheumatology Department, Faruk Medical City, Sulaymaniah, Iraq

**Keywords:** Thyrotoxic periodic paralysis, Hyperthyroidism, Hypokalemia

## Abstract

Thyrotoxic periodic paralysis (TPP) is a rare condition related to thyrotoxicosis seen predominantly in men of Asian origin. Here I present a case of a 34-year-old Chinese man who presented with sudden onset weakness of his upper and lower extremities that resolved spontaneously. His investigations revealed elevated free thyroxin (FT4), free triiodothyronine (FT3), suppressed thyroid-stimulating hormone (TSH) and hypokalemia, and he was diagnosed with thyrotoxic periodic paralysis. It is important to consider the diagnosis of TTP in patients presenting with acute onset of weakness. This report reviews the literature looking at pathophysiology, clinical features, and treatment for this rare complication of hyperthyroidism.

## INTRODUCTION

Thyrotoxic periodic paralysis (TPP) is a type of hypokalemic periodic paralysis. Hypokalemic periodic paralysis can be either primary (familial) or secondary. Secondary causes include thyrotoxicosis, hyperaldosteronism, nephritic syndrome, diabetic ketoacidosis, drugs, diuretic or laxative abuse, vomiting and diarrhea.^1^ Association of periodic paralysis and thyrotoxicosis was first described by Rosenfeld in 1902.^2^ TTP is reported more commonly in oriental Asians.^3^ TPP is characterized by transient muscle weakness that occur episodically and hypokalemia due to uncontrolled hyperthyroidism.^4^ Given its rarity and the often mild hyperthyroid symptoms, it may be missed during the initial attacks.^5^ Despite similar clinical manifestations, it is extremely important to differentiate TPP from familial hypokalemic periodic paralysis (FHPP) as treatment differ significantly.^4^

## CASE REPORT

A 34-year-old Chinese man presented to the rheumatology department of our hospital with a 1-week history of muscle weakness and pain. He was referred by the general practitioner in the company he worked in for further evaluation. The patient admitted a history of sudden onset weakness involving his upper and lower limbs 1 day prior to his presentation. The patient was well before he went to bed at 10 pm, but when he woke at about 6 am he was unable to move his upper and lower limbs; just his neck. Symptoms lasted about 1 hour, then resolved completely. The patient had physically demanding work the day before. One week earlier, the patient complained of pain and weakness in lower limbs when he woke up for urination at around 4:00 am, to the point he reached the toilet with extreme difficulty. This took about 30 minutes to resolve.

The patient attributed that to his long flight the proceeding day. Since then, he had been experiencing muscle pain mainly involving the thigh, gradually increasing in severity and later involving the arm which was also associated with stiffness and mild weakness.

On the top of musculoskeletal symptoms during the past 3 months he reported sweating, heat intolerance, palpitations, and unintentional weight loss of 6 kg despite good appetite, but did not report any changes in skin, and hair.

There was no history of preceding fever, vomiting, diarrhea, trauma or seizures. The patient denied having shortness of breath, urinary problem, abdominal pain, backache, involuntary movements, and sensory loss in any limb. There was no history of recent drug ingestion. He denied any intake of supplements, alcohol or drug abuse. He smoked 1 pack of cigarettes daily for 8 years. There was no significant past illness, and family history was noncontributory.

On examination, the patient was conscious and oriented, and looked slightly anxious. He was afebrile with a pulse of 90 beats per minute, respirations of 16 breaths per minute, and blood pressure 120/70 mm Hg. The thyroid gland was diffusely enlarged, firm, smooth, and not tender, with no audible bruit. There was no lid lag, lid retraction, or exophthalmos. Fine tremor was detected on outstretching of the hands. Cardiac, respiratory, and abdominal examination were normal. Musculoskeletal examination was unremarkable apart from quadriceps, deltoid, and biceps tenderness. Neurological examination of the upper and lower limbs revealed normal tone, proximal muscle weakness (4/5 power in the lower limbs, 4+/5 power in the upper limbs), normal distal muscle strength. Reflexes were brisk and plantar response was flexor bilaterally. There were no sensory abnormalities and the cranial nerves were intact.

Laboratory studies revealed potassium of 3.2 mmol/L (3.5–5.1 mmol/L), TSH<0.005 μIU/L (0.25–4.55 μIU/L), FT4 71.1pmol/L (12–22 pmol/L), FT3 22.6 pmol/L (3.1–6.8 pmol/L), and total creatine kinase(CK) 587 U/L(39–308 U/L). All other lab tests including full blood count, erythrocyte sedimentation rate, kidney and liver function test, blood sugar, serum calcium, serum magnesium, serum phosphorus, hepatitis serology, brucella agglutination test, lactate dehydrogenase, rheumatoid factor, and antinuclear antibodies (ANA) were normal.

His clinical presentation and laboratory abnormalities were consistent with thyrotoxic periodic paralysis. Patient was referred to endocrinologist who prescribed him propranolol 40 mg BID and Carbimazole 45 mg/day. On follow up 2 months later, the patient was subjectively well, and free from TTP attacks. His thyroid function test and serum potassium were normal.

## DISCUSSION

Periodic paralysis is a rare complication of hyperthyroidism, more common in Asian men between the second and fourth decades of life.^6^ The incidence of TPP in Chinese and Japanese thyrotoxic patients has been reported at 1.8% and 1.9%, respectively, whereas in North Americans at 0.1%–0.2%. In the Chinese, TPP occurs in 13% of male and 0.17% of female thyrotoxic patients, shown in a series published in 1967. Although thyrotoxicosis has a higher incidence in women, TPP affects men predominantly, with an overall male-to-female ratio ranging from 17:1 to 70:1.^3^

Although the exact pathophysiology of TPP is still unclear, recent data indicate that TPP results from the combination of genetics, thyrotoxicosis and environmental factors.^7^ The primary defect in TPP is intracellular shifting of potassium while potassium stores in the body remain normal. Thyroid hormones alter the permeability of plasma membrane to potassium by increasing the Na/K ATPase activity.^8^ Skeletal muscles exhibit an increase in the β adrenergic receptors, with resultant increase the Na/K ATPase activity.^3^ The Na/K ATPase pump is also activated by insulin, which may explain the link between ingestion of a high carbohydrate food load, and the paralytic attacks.^9^ The Na/K ATPase activity may be also stimulated by androgens and is inhibited by estrogens and progesterones, which may explain the predominance of the disorder among men.^9^ Genetic predisposition plays a role in the pathogenesis of TTP and may also explain the racial difference in prevalence of this condition.^8^ Mutations in the gene encoding Kir2.6, a skeletal muscle-specific Kir channel, have been found to be associated with TPP in some recent studies.^3^

The diagnosis of TPP is based on the presence of clinical and biochemical evidence of hyperthyroidism with hypokalemia in a patient with a history of paralytic attacks.^5^ Clinically, TTP is characterized by recurrent, transient episodes of muscle weakness that range from mild weakness to complete flaccid paralysis, each episode last from a few hours up to 72 h, with complete recovery in between them.^3^ Attacks commonly occur at night or early in the morning on awakening,^9^ as in our patient. Attacks are usually precipitated by strenuous exercise, high carbohydrate meals or alcohol intake.^10,11^ Proximal muscles are affected more severely than distal ones and lower extremities are affected more than the upper ones.^4^ The severity of muscle weakness correlates with the degree of hypokalemia.^4^ Mild myalgia is a complaint in less than half of patients.^3^ Deep tendon reflexes are usually diminished or absent, although normal or hyperactive reflexes may be seen in some cases. Bulbar, ocular and respiratory muscles are rarely involved. Sensory functions and cognition remain intact.^12^ Rare complications include life-threatening ventricular arrhythmias, acute respiratory failure and colonic pseudo-obstruction.^11^ Most patients with TPP have only mildly elevated serum thyroid hormone levels and only about 10% of patients may have mild thyrotoxic symptoms.^3^ Although the majority of cases of thyrotoxicosis associated with TPP are due to Graves’ disease, TPP can appear with thyrotoxicosis of any origin.^4^

During an attack of paralysis or weakness, serum potassium level is usually less than 3.0 mmol/liter and can be as low as 1.1 mmol/liter. Occasionally if the patient is at the recovery stage of the paralysis, serum potassium can be normal. Hypokalemia, may be accompanied by hypophosphatemia and hypomagnesaemia. In about two thirds of patients, serum creatine phosphokinase of muscle origin is elevated, particularly among those whose attacks are precipitated by exercise. The complication of rhabdomyolysis may occur in a severe attack.^3^

The therapy of TPP is the management of thyrotoxicosis with antithyroid drugs, radioactive iodine, or surgery.^12^ An acute episode is usually transient and may resolve even without treatment.^5^ An acute episode is treated with potassium replacement (oral or parenteral) but rebound hyperkalemia which could be potentially fatal may occur if excess potassium is supplemented as total body potassium is normal.^4^ Unlike familial hypokalemic periodic paralysis, the use of regular potassium supplementation or potassium sparing diuretics in between attacks is not justified.^11^ Restoration of euthyroidism will prevent future attacks; however, non-selective β-blockers can be used to prevent attacks until a euthyroid state is achieved.^12^ Precipitating factors such as strenuous exercise or a high carbohydrate diet should be avoided until a euthyroid state is achieved.^4^

In conclusion, TPP should be considered in all cases of acute hypokalemic paralysis, even in the absence of previous diagnosis of hyperthyroidism. TPP is typically present in young Asian men and can be the first manifestation of thyrotoxicosis. Therefore, thyroid function tests should be performed in all cases of periodic paralysis to make an early diagnosis of TPP and to start definitive treatment, as it does not occur once euthyroidism is achieved.
